# Case Report: An unclassified T cell lymphoma subtype with co-expression of TCR αβ and γ chains revealed by single cell sequencing

**DOI:** 10.3389/fimmu.2023.1184383

**Published:** 2023-05-30

**Authors:** Wei Song, Gang Wang, Cheng Wang, Lulu Liu, Liming Zhang, Ruoyu Zhang, Haixi Zhang, Keqian Shi

**Affiliations:** ^1^ Department of Radiology, The First People’s Hospital of Yunnan Province, Kunming, Yunnan, China; ^2^ School of Medicine, Kunming University of Science and Technology, Kunming, Yunnan, China; ^3^ Innovec Biotherapeutics, Inc., Beijing, China; ^4^ Department of Hematology, The First People’s Hospital of Yunnan Province, Kunming, Yunnan, China; ^5^ Yunnan Province Clinical Center for Hematologic Disease, Kunming, Yunnan, China; ^6^ Yunnan Province Clinical Research Center for Hematologic Disease, Kunming, Yunnan, China

**Keywords:** TCR - T cell receptor, T cell lymphoma, single cell, single cell RNA seq, single cell TCR sequencing

## Abstract

**Background:**

T cell lymphomas (TCL) are a group of heterogeneous diseases with over 40 subtypes. In this study, we identified a novel TCL subtype which was featured by a unique T cell receptor (TCR) presentation, α, β and γ chains were co-existing in a single malignant T cell.

**Case presentation:**

A 45-year-old male patient was diagnosed T cell lymphoma after 2-month of abdominal distension and liver enlargement. Combining histology review, PET-CT scanning and immunophenotyes, the patient was not classified to any existing TCL subtypes. To better understand this unclassified TCL case, we performed single cell RNA sequencing paired with TCR sequencing on the patient’s PBMC and bone marrow samples. To our surprise, we identified that the malignant T cells had a very rare TCR combination, by expressing two α chains, one β chain and one γ chain simultaneously. We further studied the molecular pathogenesis and tumor cell heterogeneity of this rare TCL subtype. A set of potential therapeutic targets were identified from the transcriptome data, such as CCL5, KLRG1 and CD38.

**Conclusions:**

We identified the first TCL case co-expressing α, β and γ chains and dissected its molecular pathogenesis, providing valuable information for precision medicine options for this novel TCL subtype.

## Introduction

T cell lymphomas (TCL) contains a group of heterogeneous non-Hodgkin lymphomas (NHL) with T cell origins. Therapy responses and outcomes remain poor for patients with TCL due to the absence of TCL-specific regimens (1, 2). In addition, TCL is highly heterogenous and contains over 40 subtypes according to 2022 WHO classification (3), making the diagnosis and treatment more challenging. In this study, by using single cell technology, we reported a patient with a previously unreported TCL subtype, which was featured by a unique TCR presentations (co-expressing of two α chains, one β chain and one γ chain). We deeply dissected the molecular signatures of this unique TCL subtype to better understand the disease biology and molecular pathogenesis, which can also accelerate the development of novel diagnosis and treatment strategies.

**Figure 1:**
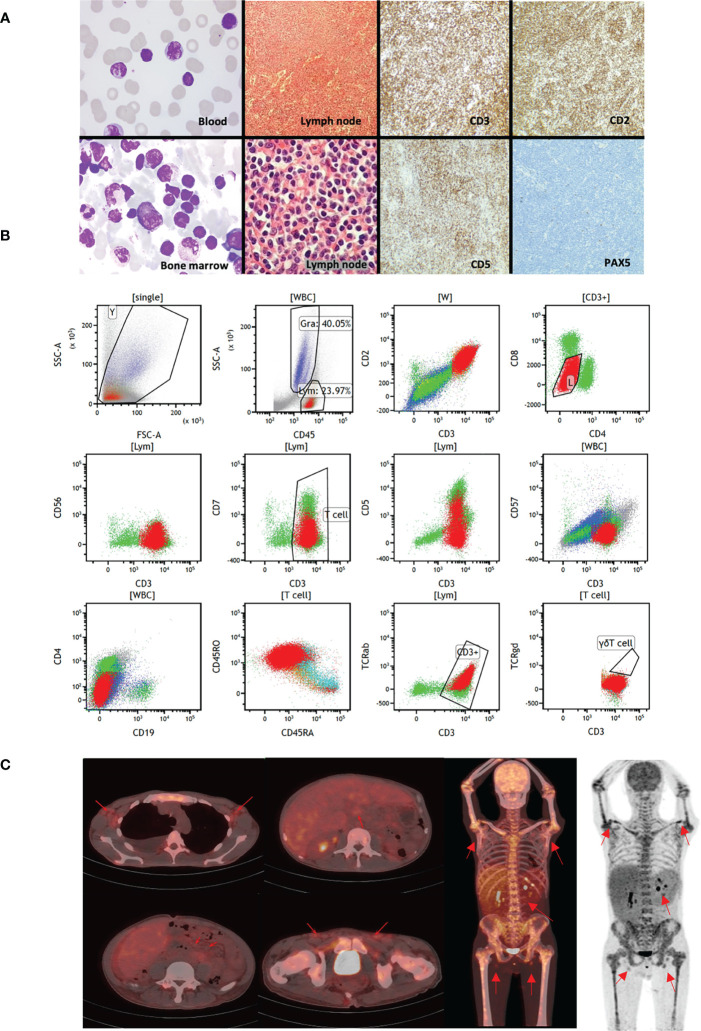
Clinical presentations of the studied patient. **(A)** The first column shows the Wright-staining of the periphery blood and bone marrow aspirate. The staining identifies small abnormal lymphocytes with round or irregular nuclei and coarse chromatin. The second column shows the H&E staining of paraffin sections of abdominal lymph node biopsies. The lymphoid tissues are hyperplasia, with uniformly small cell size, with round or irregular nuclei and coarse chromatin. The third and fourth column were immunohistochemistry of lymph nodes, suggesting CD2+, CD3+, CD5 partial+, PAX-5 Lymphatic follicle+ tumor cells. **(B)** Immunophenotypes of the malignant T cells by flow cytometry. **(C)** PET-CT scans of the patient. The red arrows indicate enlarged lymph nodes in bilateral axilla, intraperitoneal cavity, retroperitoneum and bilateral inguinal area, with increased radioactive uptake. Whole body PET images suggest that diffused radioactive uptakes are increased in the four limb bones, trunk bones, and pelvic medullary cavity.

The patient was a 45-year-old Chinese man with a history of hepatitis B. He underwent a splenectomy 6 months before TCL diagnosis due to abdominal pain. He also experienced a 2-month abdominal distension and liver enlargement. Histology review of the bone marrow aspirate identified mature but dysplasia lymphocytes with a diffused distribution, whose morphology suggested lymphoproliferative disorders (**Figure 1A**). Flow cytometry revealed a group of mature T lymphocytes which were suspected to be malignant cells with the following immunophenotypes: CD2+, CD3+, CD4-, CD8dim+, CD5dim+, CD7dim+, CD56-, CD57- and TCRαβ+ (**Figure 1B**). TCR gene rearrangement assay suggested TCR rearrangement in TCRB and TCRG but not TCRD. The patient’s lab examinations were summarized in **Table S1**. In the PET-CT examination, enlarged lymph nodes were observed in the patient’s bilateral axilla, intraperitoneal cavity, retroperitoneum and bilateral inguinal area, with increased radioactive uptake, (SUV max 2.25). Diffused radioactive uptake was increased in the four limb bones, trunk bones, and pelvic medullary cavity (SUV max 3.44). The liver was also enlarged, with increased radioactive uptake (SUV max 2.80) (**Figure 1C**). The above signs were considered to be caused by hematological malignancies. Putting the clinical evidences together, this patient did not match to any classified TCL subtype (2, 3). The patient received a 5-day E-CHOP chemotherapy as the induction treatment. The drug administration schedules were summarized in **Table S2**. The size of the patient’s liver and lymph nodes decreased during the chemotherapy, as well as the white blood cell count. However, the signs and symptoms relapsed two weeks after the induction treatment completed. Overall, the patient did not response to the standard of care well.

**Figure 2:**
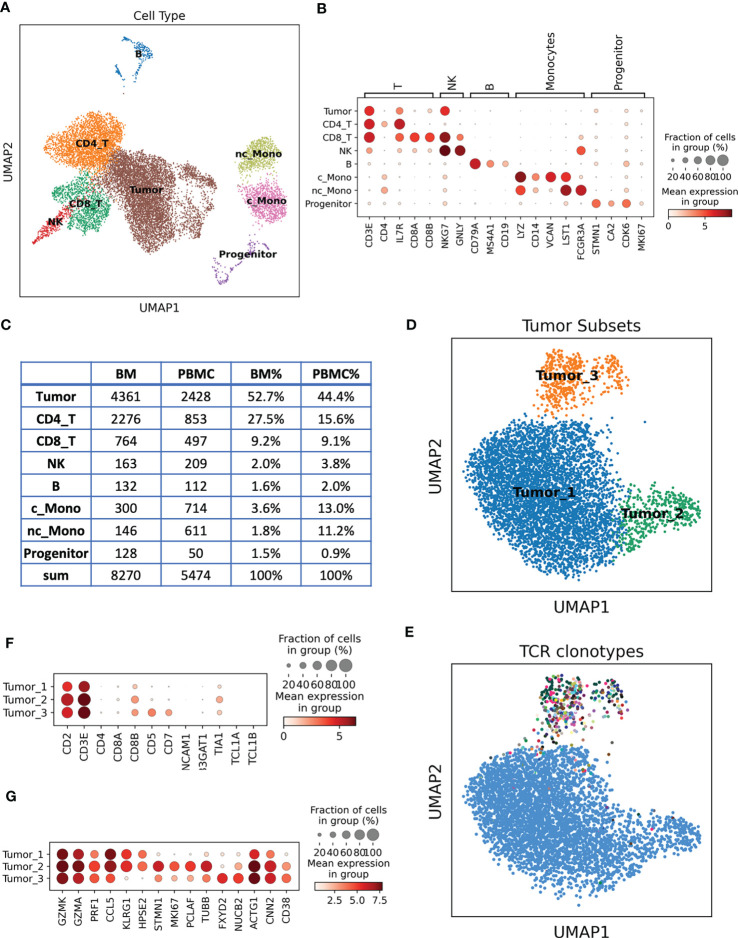
Single cell analysis of the unclassified TCL patient samples. **(A)** Cell types revealed by scRNA-seq and presented by UMAP. **(B)** Dotplot of the canonical marker genes used to annotate the cell types. Dot size represents % of cells of that cluster expressing the given gene, while color indicates the expression level of that cluster. **(C)** Cell type count and % in the BM and PBMC samples. **(D)** UMAP of re-clustered malignant T cells, cells are colored by different tumor subsets. **(E)** UMAP of malignant T cells, each color represents a distinct TCR clonotype. Tumor_1 and Tumor_2 belong to the same clonotype (blue dots), while Tumor_3 contains diverse clonotypes. **(F)** Dotplot of the marker genes used in flow cytometry analysis, single cell expression data are consistent with flow cytometry results. **(G)** Unbiasedly identified signature genes of each tumor subset.

To better understand this previously undefined T cell lymphoma subtype, we performed paired single cell RNA sequencing (scRNA-seq) and T cell receptor sequencing (scTCR-seq) on PBMC and bone marrow samples from this patient after the induction treatment. After quality control and doublet removal, 13,744 cells were retained for downstream analysis. Unsupervised clustering followed by uniform manifold approximation and projection (UMAP) enabled the clear distinction of major cell types, including tumor cells in PBMC and bone marrow. Cell types can be annotated by their canonical markers (**Figures 2A, B**, **Figure S1**): CD4 and CD8 T cells (CD3E, CD4, CD8A, CD8B); natural killer (NK) cells (GNLY, NKG7); B cells (CD19, CD79A); Hematopoietic stem and progenitor cells (STMN1, CA2, CDK6, MKI67); we also identified two monocyte subtypes: CD14+CD16- classical monocytes (c_Mono) and CD14loCD16+ nonclassical monocytes (nc_Mono). Besides these normal cells, we revealed a cluster of T cell lineage tumor cells, accounting for 52.7% and 44.4% of the cell population in BM and PBMC, respectively (**Figures 2A, C**).

We next re-clustered the tumor cells to reveal the tumor molecular signatures and heterogeneity. Three tumor subsets have been identified (**Figure 2D**): Tumor_1 is the major subset, accounting for 84.8% of the tumor cells, Tumor_2 and Tumor_3 were relative smaller tumor subsets, account for 7.2% and 8.0%, respectively. By integrating the TCR information, we found that Tumor_1 and Tumor_2 cells shared the same TCR sequences, suggesting that they arose from the same tumor ancestor cell and evolved into two branches (**Figure 2E**). Tumor_3 had a distinct TCR profile compared to Tumor_1 and Tumor_2 (**Figure 2E**). We surprisingly found that the Tumor_1 and Tumor_2 cells had a unique TCR presentation, a single tumor cell expressing two α chains (TRAV27 + TRAJ44 + CADTGTASKLTF and TRAV12-1 + TRAJ47 + CVVIYGNKLVF), one β chain (TRBV10-2 + TRBJ2-2 + CASSANTGELFF) and one γ chain (TRGV9 + TRGJ1 + CALVAVGREEFYYKKLF), but no δ chain. The four TCR chains had comparable expression levels according to their TCR V gene expressions (**Figure S2**). T cells usually express either a pair of αβ chains or a pair of γδ chains, it is very rare to observe the co-expressing of αβ and γδ chains in a single T cell (4, 5). To our knowledge, here, we identified the first case of T cell lymphoma with α, β and γ chains presenting together in a single tumor cell. The co-expressing of α, β and γ chains indicated a unique putative cell of origin of these tumor cells, the ancestor cell might be a combined αβ and γδ T cell. This special TCR presentation can be related to the pathogenesis of this rare tumor subtype, explaining the poor prognosis of the patient. The Tumor_3 cluster were not monoclonal expanded, the biggest clonotype in Tumor_3 contained 48 cells (13.8%). We further inferred the chromosome copy number variation (CNV) using scRNA-seq data, we identified Chr5 duplications in Tumor_1 and Tumor_2 but not in Tumor_3 (**Figure S3**). Taking together the UMAP distance, CNV and TCR information, we hypothesized that Tumor_3 might be a pre-malignant T cell cluster.

scRNA-seq provided rich information for us to investigate the molecular signatures of the tumor cells and propose precision therapeutic strategies for the patients. Aligning with the flow cytometry data, we confirmed that the tumor cells were CD2, CD3 positive, CD8A negative but CD8B positive. CD5 and CD7 were only presented at Tumor_3 cells, consistent with the partial positive observation in the flow cytometry data (**Figure 2F**, **Figure S4**). We next unbiasedly identified the signature genes of each tumor subset (**Table S3**-**S5**, **Figure 2G**). Tumor_1 and Tumor_2 displayed cytotoxic T cell phenotypes by expressing a cytotoxic module, including GZMK and GZMA, PRF1 etc., which supported their putative CD8 T cell and γδ T cell origins (6). Several potential therapeutic target genes were also identified in Tumor_1 and Tumor_2 (**Figure 2G**). For example, CCL5, abnormal expression and activity of CCL5/CCR5 axis have been found in hematological malignancies and solid tumors. Therapeutic strategies targeting CCL5/CCR5 might be effective for these tumor cells (7). KLRG1, KLRG1 were upregulated in human tumor samples and potentially contributing to adaptive resistance (8), KLRG1 blockade was found to be effective at slowing tumor growth (9). Tumor_2 shared molecular similarities with Tumor_1, but it was featured by high expression of proliferation markers such as MKI67, STMN1, suggesting they are the actively expansion tumor cells. Tumor_3 were the pre-malignant T cell cluster with a distinct expression profile (**Figure 2G**, **Table S5**). CD38 were one of the marker genes of Tumor_3, and it is a demonstrated target for immunotherapeutic approaches of multiple myeloma (10). Collectively, we established a molecular subtyping scheme of this rare TCL based on the transcriptome of malignant T cells and provided vast information of new treatment options for the patient.

In conclusion, we report the first case of T cell lymphoma with TCR α, β and γ chains co-expressing, and identified the unique gene expression profiling and heterogeneity of the tumor cells, paving the way for future investigation and treatment of this novel TCR subtype.

## Methods

### Patient recruitment and ethics statement

This study was approved by the Ethics Committee of The First People’s Hospital of Yunnan Province, China (#KHLL2022-KY005). The recruited patient gave informed consent at hospitalization.

### Clinical examinations

Nonenhanced whole-body 18F-FDG PET/CT imaging was obtained using the scanner of Ingenuity TF (Koninklijke Philips N.V., Netherlands). The fluorine 18 (18F)-labeled glucose analog 18F-FDG was injected as the PET radiotracer. The acquisition range was from the top of the head to the bottom of the foot. All PET images were reconstructed using an iterative algorithm with attenuation correction on the scanner.

Abdominal lymph node biopsies were obtained for histology analysis. All specimens were fixed in formal saline, processed, and embedded in paraffin wax. Hematoxylin-eosin sections from one to six paraffin sections were examined. Immunohistochemical analysis was performed on 3-𝜇m-thick formalin-fixed/paraffin-embedded sections. The following antibodies were used for staining: CD20, CD79a, CD2, CD3, CD5, CD10, CD21, CD38, BCL-2, MPO, Mum-1, CyclinD1, PAX-5, Ki-67, CD56, TIA-1, TdT, cd34, EBER (Maxim Biotech, China).

Peripheral blood and bone marrow samples were collected for smear cytology for Wright staining. The staining was performed with following steps: 1) Add Wright-Giemsa Solution A (~ 0.5~0.8ml) to the smear and stain for 1 min. 2) Add Wright-Giemsa Solution B (2~3 volumes of Solution A) onto Solution A and mix thoroughly then stain for 5~10mins. 3) Rinse with water gently, dry and examine the slide using a microscope.

The antibodies used in the flow cytometry analysis were CD7, CD45, CD38, TCRgd, CD34, CD19, CD3, CD2, CD117, CD56, CD5, CD3, CD13, CD33, TCRab, CD16, TRBC1, CD4, CD8, CD45RA, CD45RO, CD57, CD94 (BD Bioscience, US). The cells were washed in phosphate-buffered saline (PBS) and stained with a cocktail of cell surface antibodies for 20 min. Lysing solution (BD Bioscience, US) was then added to remove red blood cells. The cells were then washed and resuspended in PBS and analyzed by flow cytometry (FACS Canto, Bioscience, US).

TCR rearrangements were detected by IdentiClone^®^ TCRB + TCRG Gene Clonality Assay following the manufacturer’s instructions (Invivoscribe, San Diego, US)

### Single cells RNA and TCR sequencing

The single cell experiment were performed as previously described (11). Briefly, the patients’ mononuclear cells were isolated from whole blood and bone marrow and resuspended with freezing medium. On the date of experiment, the cells were thaw using a water bath at 37°C and loaded into Chromium microfluidic chips and barcoded within a 10X Chromium Controller (10X Genomics, US). For transcriptome, procedures were performed with reagents: Chromium Next GEM Single Cell 5’ Reagent Kits v2 (Dual Index) (10X Genomics, PN-1000263). TCR enrichment was carried out using the Chromium Single Cell Human TCR Amplification Kit (10X Genomics, PN-1000252) for αβ transcripts, or customer primers for γδ TCR transcripts (12). The sequences of the customer primers for γδ TCR were listed. Human γδ T cell mix1: Forward: 5’-GATCTACACTCTTTCCCTACACGACGC-3’. Reverse Outer Primers: 5’-CTTCATATTTACCAAGCTTGACAG-3’ and 5’-GGTGTTCCCCTCCTGG-3’. Human γδ T Cell Mix 2: Forward: 5’-GATCTACACTCTTTCCCTACACGACGC-3’, Reverse Outer Primers: 5’-GATGACAATAGCAGGATCAAAC-3’ and 5’-CCCAGAATCGTGTTGCT-3’. All the libraries were sequenced on the NovaSeq 6000 platform (Illumina, US).

### Single cell data analysis

Raw sequencing data were processed by Cell Ranger version 7.0.1 (10X Genomics) to generate gene expression matrix and assemble TCR sequences with human GRCh38 reference genome. scRNA-seq data analysis were performed with Scanpy (13). In quality control step, cells were filtered by the following criteria: 1) mitochondrial abundance < 15%, 2) minimum gene detected > 500, 3) Maximum UMI < 40000. Potential doublets were detected and removed by Scrublet (14). Principal component analysis (PCA) was applied on the highly variable genes. Batch effects were corrected using Harmony (15). Cells were then projected and visualized in 2D dimensions using uniform manifold approximation and projection (UMAP. Cells were clustered by Leiden algorithm implemented in Scanpy. TCR data was analyzed with Scirpy (16) and customized scripts. R package InferCNV was used to infer copy number variation (https://github.com/broadinstitute/infercnv).

## Data availability statement

The sequencing data was deposited at Gene Expression Omnibus (GEO) under accession no. GSE222200.

## Ethics statement

The studies involving human participants were reviewed and approved by ethics committee of The First People’s Hospital of Yunnan Province, China. The patients/participants provided their written informed consent to participate in this study. Written informed consent was obtained from the participant/patient(s) for the publication of this case report.

## Author contributions

RZ, KS, HZ, WS, and GW initiated the project and designed the experiment. WS, HZ, LL, and LZ performed the majority of experiments. HZ and KS involved in patient recruitment and sample collection. WS and GW analyzed the CT scanning data. RZ performed the bioinformatics work. RZ, KS, WS, HZ, GW, and CW interpreted the data and results. RZ wrote the initial draft with all authors providing input on the manuscript. All authors contributed to the article and approved the submitted version.
