# Supplementary figures and images for: Case Report: An unclassified T cell lymphoma subtype with co-expression of TCR αβ and γ chains revealed by single cell sequencing
Source: Front Immunol. 2023 May 30;14:1184383. doi: 10.3389/fimmu.2023.1184383 (PMC10266344; doi:10.3389/fimmu.2023.1184383)

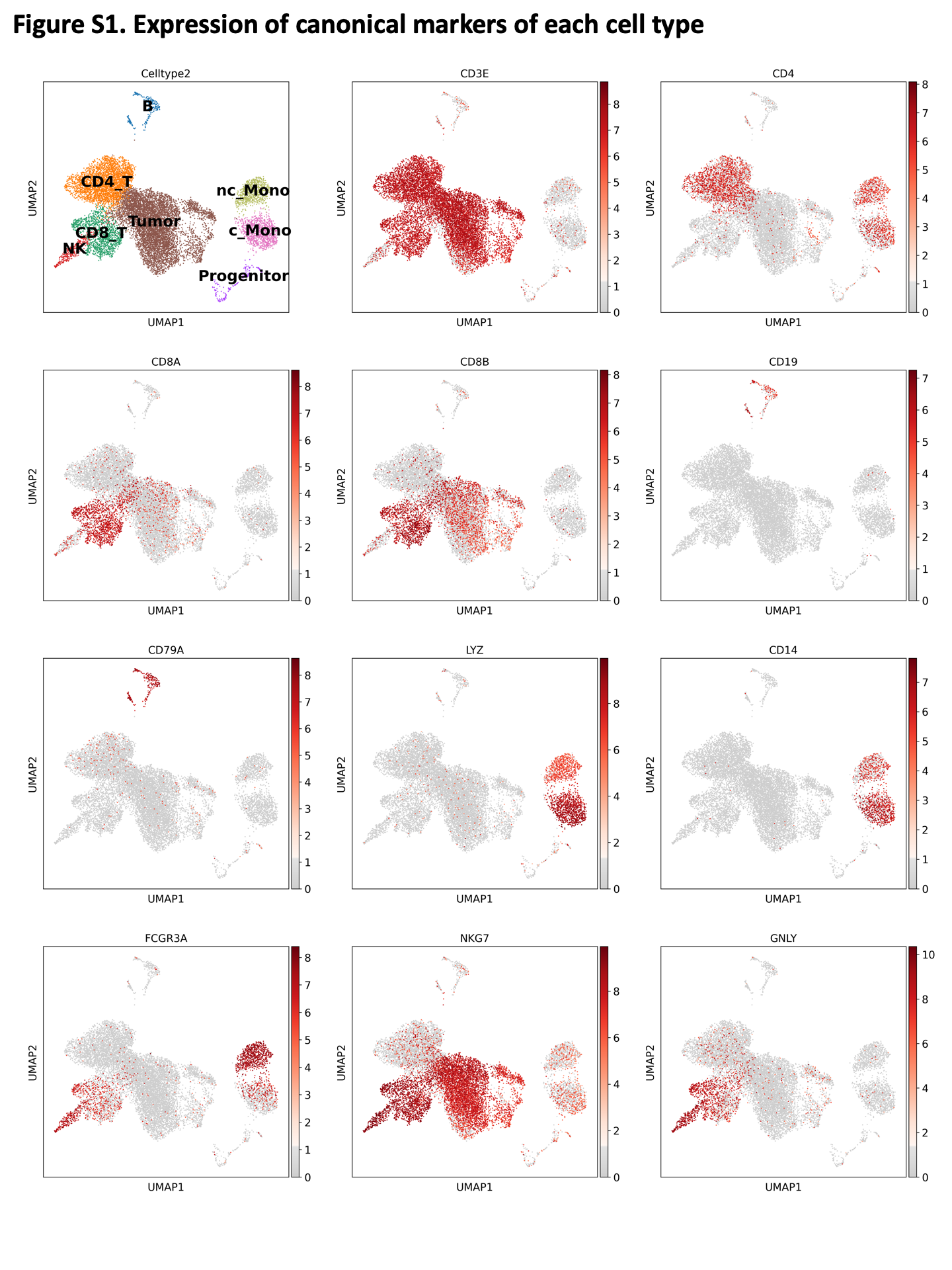

Supplement: Supplementary file 6 [file Image_1.tiff]

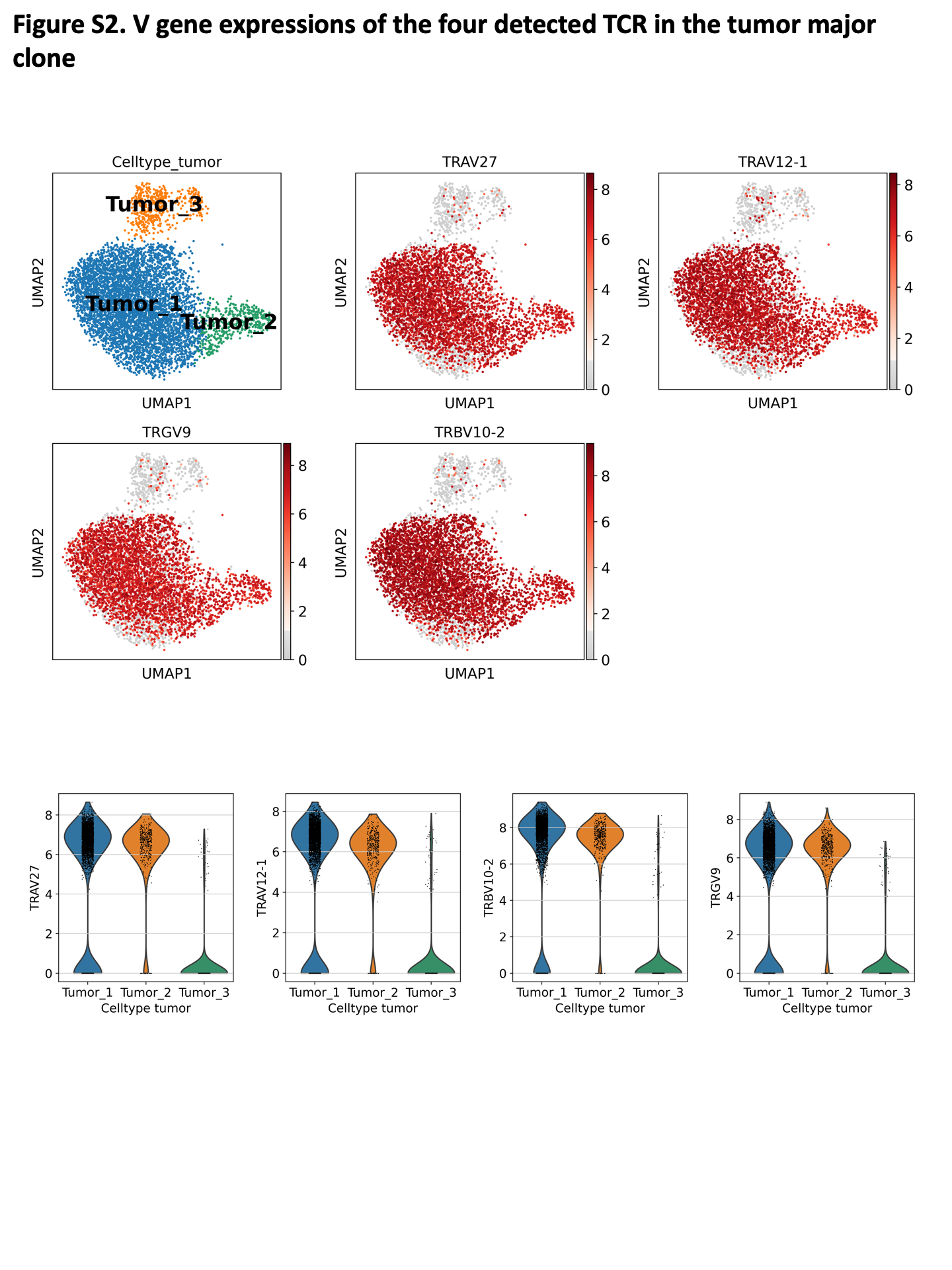

Supplement: Supplementary file 7 [file Image_2.tiff]

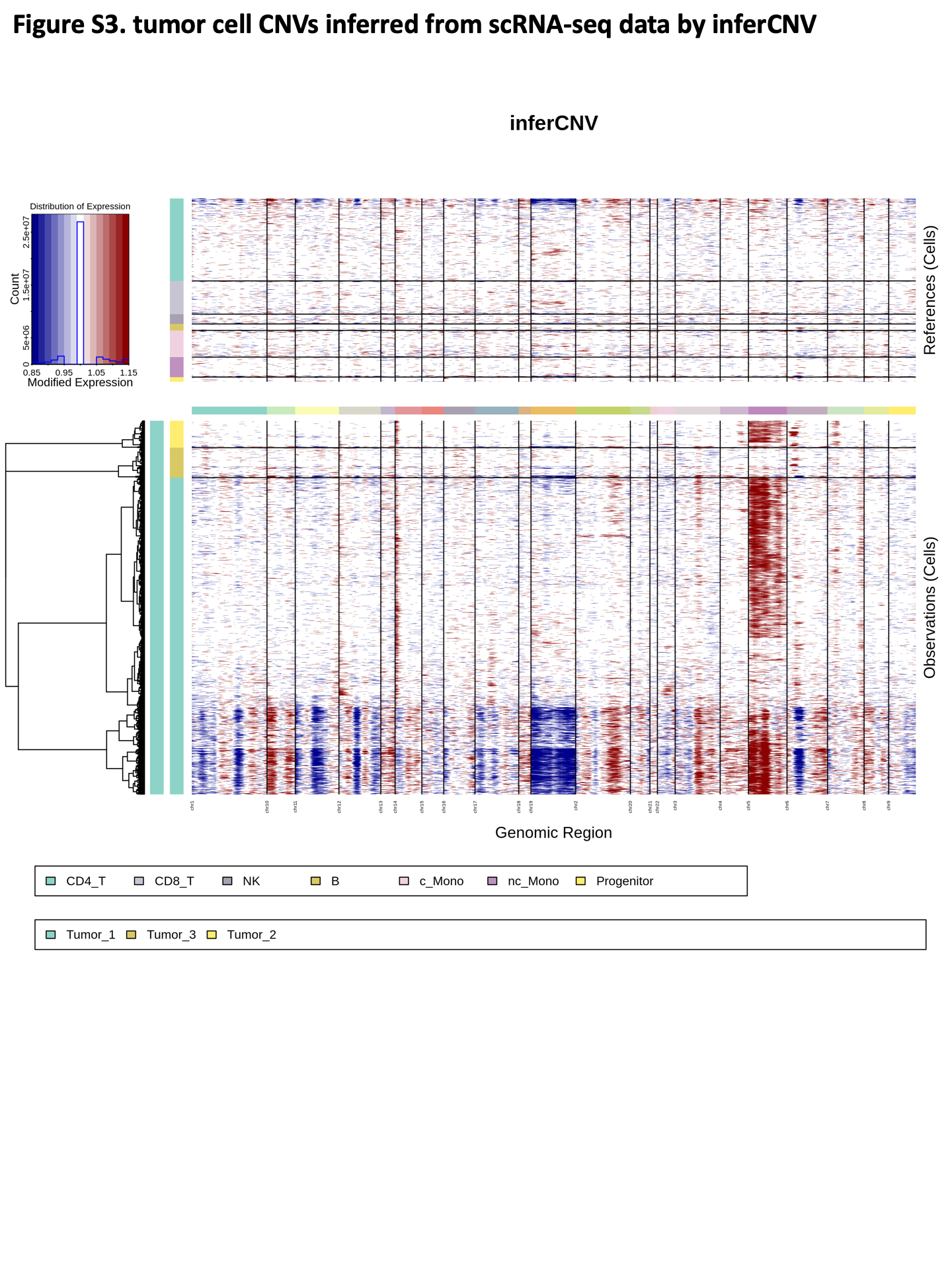

Supplement: Supplementary file 8 [file Image_3.tiff]

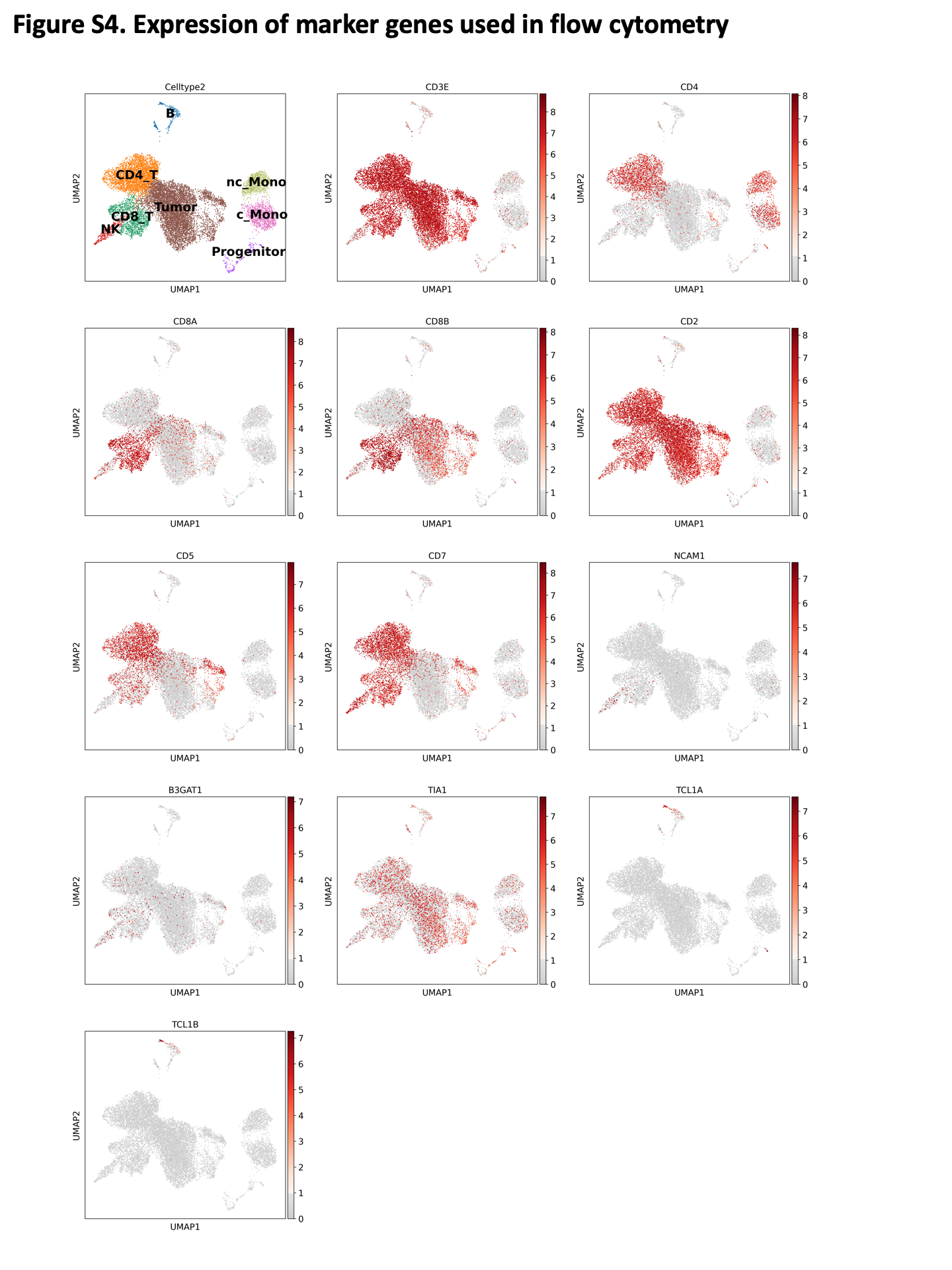

Supplement: Supplementary file 9 [file Image_4.tiff]
